# Supplementary material for: Identification of photoperiod-induced specific miRNAs in the adrenal glands of Sunite sheep (Ovis aries)
Source: Front Vet Sci. 2022 Jul 22;9:888207. doi: 10.3389/fvets.2022.888207 (PMC9354845; doi:10.3389/fvets.2022.888207)
Supplement: Supplementary file 1 [file Data_Sheet_1.ZIP › Supplementary file/miRDeep2 file.zip!/miRDeep2 file/Novel_Structure/SP42A2/Novel_492.pdf]

Provisional ID	:	NC_019458.2_2854
Score total	:	1.9
Score for star read(s)	:	-1.3
Score for read counts	:	0
Score for mfe	:	1.6
Score for randfold	:	1.6
Score for cons. seed	:	
Total read count	:	107
Mature read count	:	107
Loop read count	:	0
Star read count	:	0

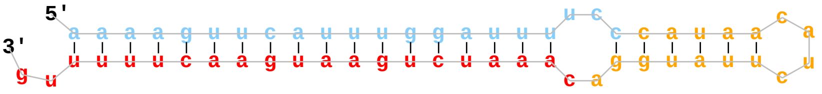

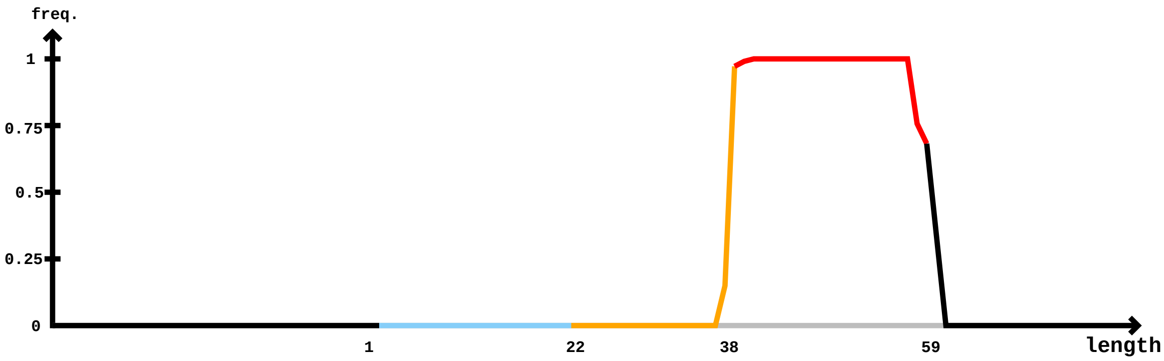

Star   Mature

```
5'- cccuaaaccauguaauaucaauugaguuggcaaaaaguucauuuggauuuucccauaacaucuuauggacaaaucugaaugaacuuuuugaccaacccagcagaaucuaga   -3'    exp
    .................((..(((.(((((((((((((((((((((((((..((((((....))))))..)))))))))))))))))))).))))).)))..)).......    reads    mm    sample
    ....................................................................acaaaucugaaugaacuuuu.......................    2        0     seq
    ....................................................................acaaaucugaaugaacuuuuug.....................    4        0     seq
    ....................................................................aAaaaucugaaugaacuuuuuga....................    10       1     seq
    .....................................................................Aaaaucugaaugaacuuuu.......................    23       1     seq
    .....................................................................caaaucugaaugaacuuuu.......................    1        0     seq
    .....................................................................caaaucugaaugaacuuuuu......................    7        0     seq
    .....................................................................Aaaaucugaaugaacuuuuug.....................    57       1     seq
    ......................................................................aaaCcugaaugaacuuuuuga....................    2        1     seq
    .......................................................................aaucugaaugaacuuuuu......................    1        0     seq
```
